# Deep learning can yield clinically useful right ventricular segmentations faster than fully manual analysis

**DOI:** 10.1038/s41598-023-28348-y

**Published:** 2023-01-21

**Authors:** Julius Åkesson, Ellen Ostenfeld, Marcus Carlsson, Håkan Arheden, Einar Heiberg

**Affiliations:** 1 Clinical Physiology, Department of Clinical Sciences Lund, Lund University, Skåne University Hospital, Lund, Sweden; 2 Department of Biomedical Engineering, Faculty of Engineering, Lund University, Lund, Sweden

**Keywords:** Image processing, Machine learning, Software, Magnetic resonance imaging

## Abstract

Right ventricular (RV) volumes are commonly obtained through time-consuming manual delineations of cardiac magnetic resonance (CMR) images. Deep learning-based methods can generate RV delineations, but few studies have assessed their ability to accelerate clinical practice. Therefore, we aimed to develop a clinical pipeline for deep learning-based RV delineations and validate its ability to reduce the manual delineation time. Quality-controlled delineations in short-axis CMR scans from 1114 subjects were used for development. Time reduction was assessed by two observers using 50 additional clinical scans. Automated delineations were subjectively rated as (A) sufficient for clinical use, or as needing (B) minor or (C) major corrections. Times were measured for manual corrections of delineations rated as B or C, and for fully manual delineations on all 50 scans. Fifty-eight % of automated delineations were rated as A, 42% as B, and none as C. The average time was 6 min for a fully manual delineation, 2 s for an automated delineation, and 2 min for a minor correction, yielding a time reduction of 87%. The deep learning-based pipeline could substantially reduce the time needed to manually obtain clinically applicable delineations, indicating ability to yield right ventricular assessments faster than fully manual analysis in clinical practice. However, these results may not generalize to clinics using other RV delineation guidelines.

## Introduction

Right ventricular (RV) delineations are of high clinical importance for providing prognostic markers such as end-diastolic volume (EDV), end-systolic volume (ESV) and RV ejection fraction (EF)^1^. However, both manual and automated delineations can be challenging since the RV is characterized by indistinct borders, trabeculations^2^, and a general complex shape variability^3^. Consequently, manual delineations may vary largely between observers and be time-consuming to obtain^4^.

Automated and semi-automated methods for RV-specific segmentation^5–9^ as well as full cardiac segmentation^10–15^ have recently been largely based on Convolutional Neural Networks (CNNs), but studies reporting on the clinical benefit of such methods are scarce^16, 17^.

The optimal automated RV delineation method produces RV delineations of clinically useful quality faster than manual delineations. We define clinically useful delineations as those that can be used clinically to determine volumes without any manual intervention. Albeit there is a wide range of existing CNN-based methods for RV segmentation, there is to our knowledge no previous study that has fully validated the aspect of clinical time reduction. Delineation times between an observer and a deep learning-based commercial cardiac segmentation software have been compared^17^, but the time for performing corrections of inadequate delineations has not previously been taken into account, even though RV delineations by contemporary deep learning methods still need to be verified by expert observers^1^.

Therefore, the aim of this study was to develop a CNN-based RV segmentation pipeline and to ensure its clinical applicability by validating its ability to reduce the time for obtaining delineations of clinically adequate quality.

## Methods

**Figure 1:**
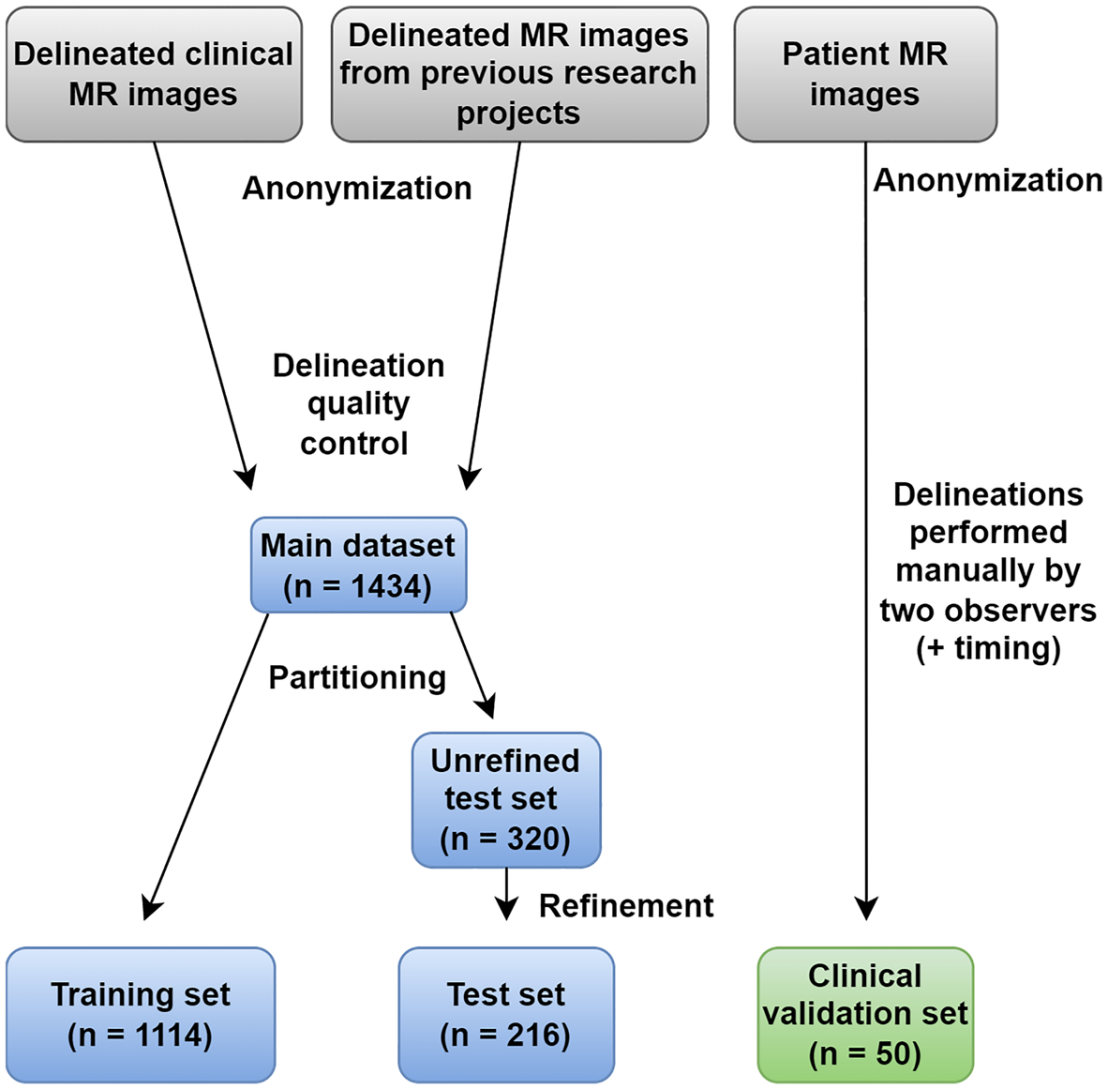
The data inclusion and data curation process. The refinement refers to the process of removing subjects with inadequate delineations in some timeframe.

We included previously delineated RV data from Skåne University Hospital, Lund, Sweden and from a wide range of medical research projects carried out by the Lund Cardiac MR Group, Lund University, Lund, Sweden. The dataset collected for developing and evaluating the pipeline was denoted as the *main dataset*, and the dataset collected for the validation of the pipeline's clinical benefits as the *clinical validation set* (CVS). Figure 1 shows an overview of the data inclusion and curation process. Complete data anonymization was carried out before inclusion using Segment^18^. Throughout the study, the quality of delineations was assessed by two observers (O1 and O2, both certified at CMR level 3)*.*

### Main dataset

The main dataset was assembled by combining short-axis CMR examinations from clinical scans collected between 2019 and 2020 (81%) with short-axis CMR data from previous research projects collected between 2004 and 2020 (19%)^19–21^.

Eighty percent of the subjects in the main dataset were acquired using scanners from Siemens, nineteen percent from Philips and the remaining from General Electric. Twenty-four percent of the research study scans were from healthy volunteers (normal individuals and athletes), and the remaining scans were from patients. Additional characteristics of the main dataset can be found in Supplementary Methods S1.

Each included CMR examination consisted of a time resolved collection of short-axis images paired with delineations of the RV endocardium in either or both the end-diastolic (ED) and end-systolic (ES) timeframes. For the main dataset, all delineations were performed by experienced physicians or MD-PhD students. In the latter case, the quality and agreement with consensus guidelines^22^ of the delineations were verified by experienced physicians.

Before inclusion, all delineations were subject to a quality control performed by one of the observers, to guarantee adequate concordance with the consensus guidelines^22^ and hence suitability for inclusion. Subjects with complex congenital heart defects (e.g. situs inversus, transposition of the great arteries, and univentricular hearts) were excluded. Subjects that only had RV delineations in non-short-axis images were excluded. The quality control reduced the initial 2490 examinations to 1693 examinations, corresponding to 1434 unique subjects. A single subject could have had several examinations.

The main dataset (n = 1434) was partitioned into a training set (n = 1114) and a test set (n = 320). Some examinations had delineations that were approved only in one of the two timeframes (ED or ES) during the quality control. From these examinations, the timeframes with approved delineations were used for the training set. For the test set, only examinations with delineations approved during the quality control in both ED and ES were included. Due to this, 104 subjects were excluded, leaving 216 subjects in the refined test set (TS).

To assess generalizability, the trained pipeline was applied to the Automatic Cardiac Diagnosis Challenge (ACDC) testing dataset and externally validated using the online challenge evaluation platform^23^. This dataset consists of short-axis CMR images of 10 healthy and 40 pathological subjects^23^. To further validate the segmentation precision of the pipeline, a scan-rescan assessment was performed using CMR images of 10 additional healthy subjects from two different scanning occasions. For 3 of the subjects, the rescans were performed using different scanners.

### Pipeline

**Figure 2:**
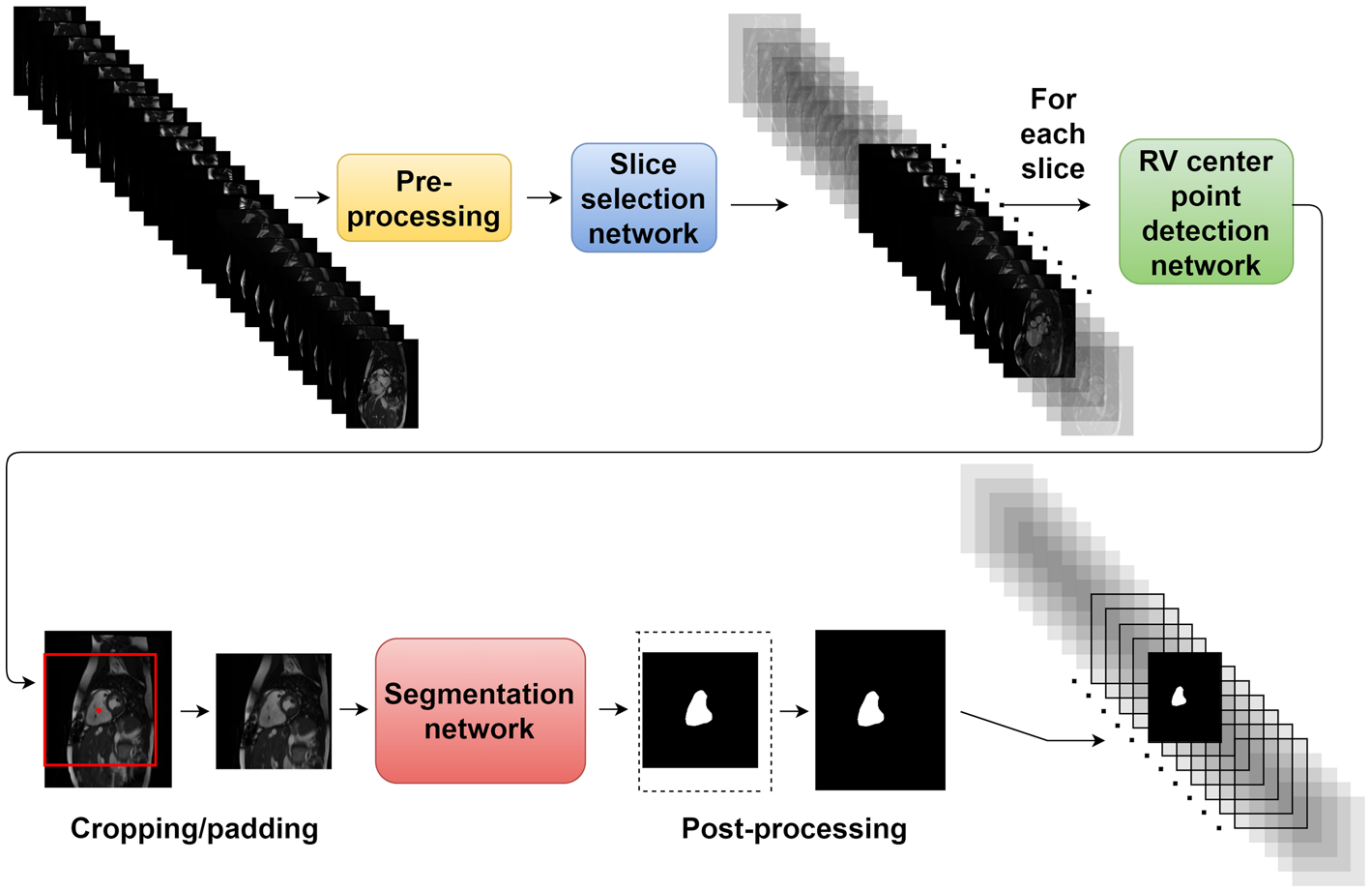
A flowchart of the pipeline. An input timeframe is pre-processed and inserted into a slice selection network that selects slices containing RV cross-sections. The RV center point is then detected in each selected slice by the RV center point detection network and used to crop (or pad) each slice around the RV before insertion into the segmentation network. This yields a segmentation of the RV, that is then inversely padded or cropped to match the original slice size.

A flowchart of the pipeline developed for this study can be seen in Fig. 2. The required input to the pipeline was a stack of short-axis images in one timeframe and its pixel spacing (the distance between pixels, in mm). The input timeframe was pre-processed by slice-wise re-sampling to the median pixel spacing of the training set (1.07 × 1.07 mm) through bilinear interpolation. After pre-processing, three subsequent CNNs were applied to the timeframe. The purpose of the two first CNNs was to handle the size variability of clinical CMR images and robustly pre-process the input data, making the third CNN (the segmentation CNN) implementable in a clinical setting. Details on each used CNN architecture are provided in Supplementary Methods S2.

The first CNN (the slice selection network) selected each slice of the input timeframe that contained a cross-section of the RV, through binary classification. The first and last selected slice were used to mark the boundaries of the RV in the slice direction. This was done in concordance with the method used by Berggren et al.^24^.

Each slice within the selected bounds of the RV was inserted into the second CNN (the RV center point detection network) that generated the coordinates of the center point of the RV cross-section in each slice. The RV center points were used to crop (or zero-pad) each slice to 256 × 256 pixels, giving all slices the same field of view and a centered RV. Each such slice was then subject to Z-score intensity normalization and inserted into the third CNN (the segmentation network) that generated a binary mask for each slice, where the cross-section of the RV endocardium was the foreground. Each generated segmentation mask was then inversely padded or cropped back to the original size of the input image, using the RV center point for positioning. As a post-processing step, only the largest connected component in each mask was taken as the RV and other components were removed.

### Overview of CNN training and pipeline evaluation

The three CNNs were trained separately using differently pre-processed versions of the training set with different ground truth definitions that matched the respective subtask of the given CNN (see Supplementary Methods S3). For all three CNN types, hyperparameter optimization was carried out using grid search five-fold cross validation on the training set. The optimized sets of hyperparameters were then used to train the final models on the full training set. Training details can be found in Supplementary Methods S3.

A performance evaluation of the final pipeline was carried out on the refined test set (TS). Although quality was controlled by a level 3 CMR reader, some of the delineations in TS were performed by non-level 3 readers. Therefore, the TS delineations were not used for validating the clinical benefits of the pipeline, but only for initial pipeline evaluation. The results from this can be found in Supplementary Results S4.

### Validation of clinical benefit using the clinical validation set (CVS)

The clinical validation set (CVS) was collected from fifty consecutive and unselected short-axis CMR examinations from clinical scans at Skåne University Hospital. Each examination was from a unique subject. The observers were blinded to all clinical and diagnostic information.

Automated delineations in ED and ES for all subjects of the CVS and TS were first generated using the pipeline. To define whether the delineations were of clinically adequate quality, O1 (expert core-lab reader^25, 26^) performed a visual rating of the overall quality of the automated delineations in both ED and ES for each subject according to a three-level modified Likert scale of decreasing delineation quality as A (sufficient for clinical use), B (needing minor corrections) or C (needing major corrections) for both the CVS and TS. Level A delineations were sufficiently close to fulfilling the consensus guidelines to likely not affect diagnosis. Level B and C delineations both deviated from the consensus guidelines enough to likely affect diagnosis but required two different levels of effort for correction. Examples of these ratings can be found in Supplementary Videos S6,S7,S8 and Supplementary Methods S5.

An assessment of the time consumption for obtaining delineations of clinically adequate quality was conducted on the CVS by O1 and O2. To do this, the delineation runtimes for the pipeline on an NVIDIA Quadro T1000 GPU were measured. Then, O1 and O2 performed timed, manual corrections of the automated delineations that had been rated as B or C. In Segment^18^, the generated contours are displayed using spline points, which allows for fine adjustments to be made to specific parts of the delineation. Lastly, the two observers each performed timed, manual delineations without prior automated delineations and according to the consensus guidelines^22^, for all subjects in the CVS. Reference volumes were computed as the mean volumes between observers. All measured times were for delineating both the ED and ES timeframes.

### Statistics

Normality of distributions were assessed through the Anderson–Darling test. Bias was assessed according to Bland–Altman analysis^27^ for both volume and ejection fraction (EF), and Spearman’s rank correlation coefficients (r) were used for comparative analyses. Relative bias was expressed as percentages of mean reference volumes. Two-sided Wilcoxon signed rank tests were used for the comparison between delineation times. Two-sample F-tests were used for determining the significance of differences in variabilities (variances). A two-sided *p* value lower than 0.05 was considered statistically significant. Segmentation performances were evaluated using Dice score (Sørensen-Dice coefficient^28^) and Hausdorff distance. MATLAB R2019a or R2021a (Natick, Massachusetts: The MathWorks Inc., 2019 and 2021) were used for all statistical analyses.

### Ethics declarations

The usage of data from clinical routine was waived by the Swedish Ethical Review Authority (Dnr 2021-03583). The usage of research study data was approved by the Regional Ethical Committee in Lund (EPN Dnr 621/2004, 2010/114, 2010/248, 2011/777, 2010/55, 741/2004 and 269/2005). All methods were performed in accordance with the Declaration of Helsinki and the guidelines and regulations set forth by the Swedish Ethical Review Authority.

## Results

For the clinical validation set (CVS), the mean ± standard deviation (SD) of reference volumes of EDV were 190.5 ± 62.8 ml and of ESV 94.0 ± 41.0 ml. For O1, EDV was 188.8 ± 61.1 ml, ESV 98.9 ± 40.8 ml, and EF 48 ± 11%. For O2, EDV was 192.3 ± 65.3 ml, ESV 89.0 ± 41.8 ml, and EF 54 ± 11%. Performance evaluations of the full pipeline carried out on the refined test set (TS) are presented in Supplementary Results S4.

For the CVS, the mean (± SD) volumetric Dice scores between the pipeline and delineations by O1 were 0.91 ± 0.02 for ED and 0.87 ± 0.04 for ES, and for delineations by O2 the Dice scores were 0.91 ± 0.02 for ED and 0.88 ± 0.03 for ES. The mean (± SD) volumetric Hausdorff distances between the pipeline and delineations by O1 were 8.47 ± 1.32 for ED and 7.71 ± 0.96 for ES, and for delineations by O2 the Hausdorff distances were 8.11 ± 1.36 for ED and 7.30 ± 1.22 for ES. Between O1 and O2, the Dice scores were 0.91 ± 0.03 for ED and 0.86 ± 0.04 for ES, and the Hausdorff distances were 8.53 ± 1.29 for ED and 7.66 ± 1.26 for ES.

**Figure 3:**
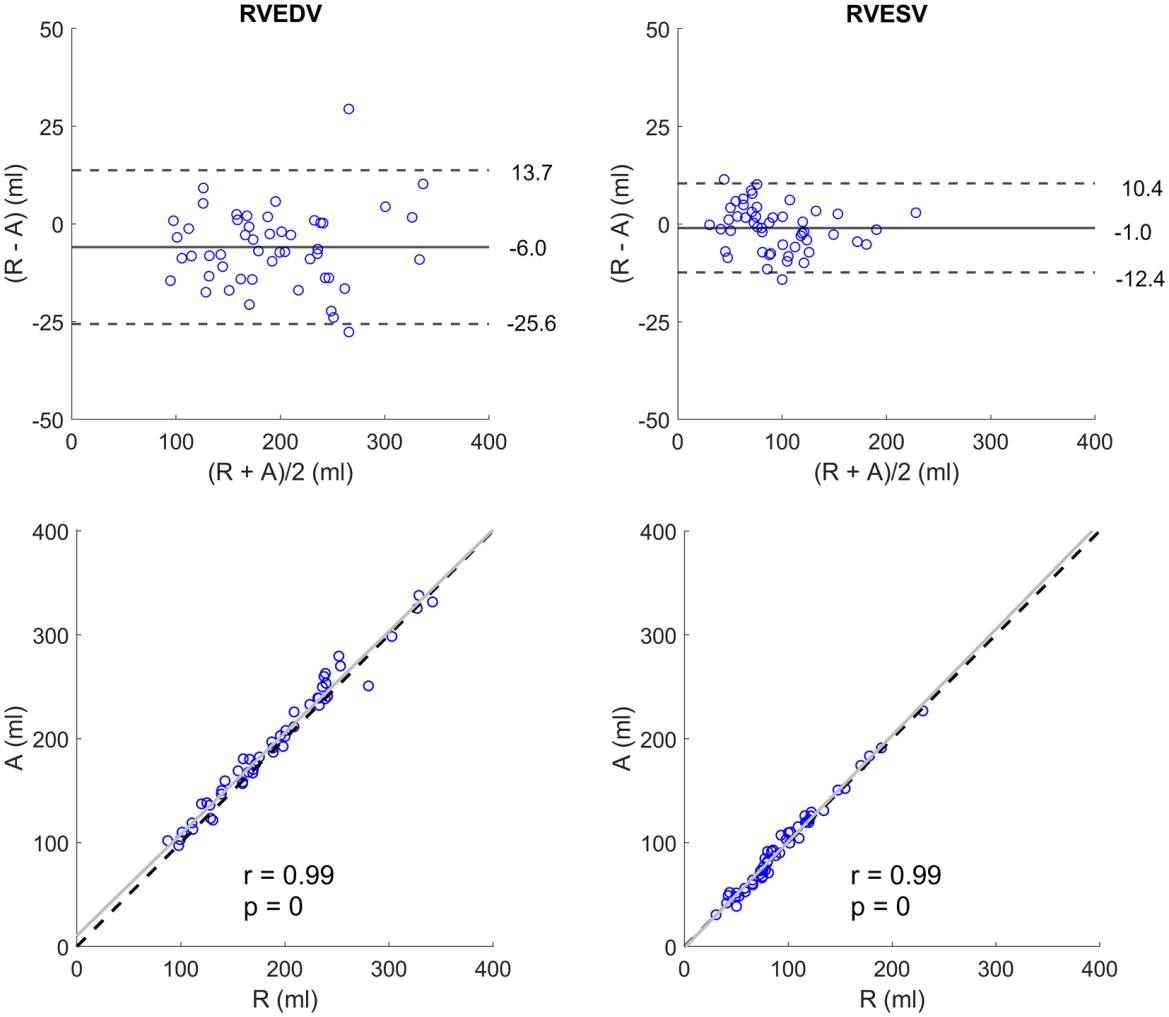
Bland–Altman and correlation plots between the pipeline’s automated (A) and the reference (R) RVEDV (left column) and RVESV (right column) on the clinical validation set (CVS). The Bland–Altman plots contain bias (full lines) and limits of agreement (± 1.96 SD, dashed lines). The correlation plots contain identity lines (black, dashed lines), least squares lines (grey, full lines), Spearman’s rank correlation coefficients (r), and corresponding *p* values.

The absolute and relative bias between the pipeline and the reference volumes was − 6.0 ± 10.0 ml (− 3 ± 5%) for EDV, − 1.0 ± 5.8 ml (− 1 ± 6%) for ESV, and 1 ± 4% for EF, and the r value was 0.99 (*p* < 0.0001, n = 50) for both timeframes (Fig. 3).

**Figure 4:**
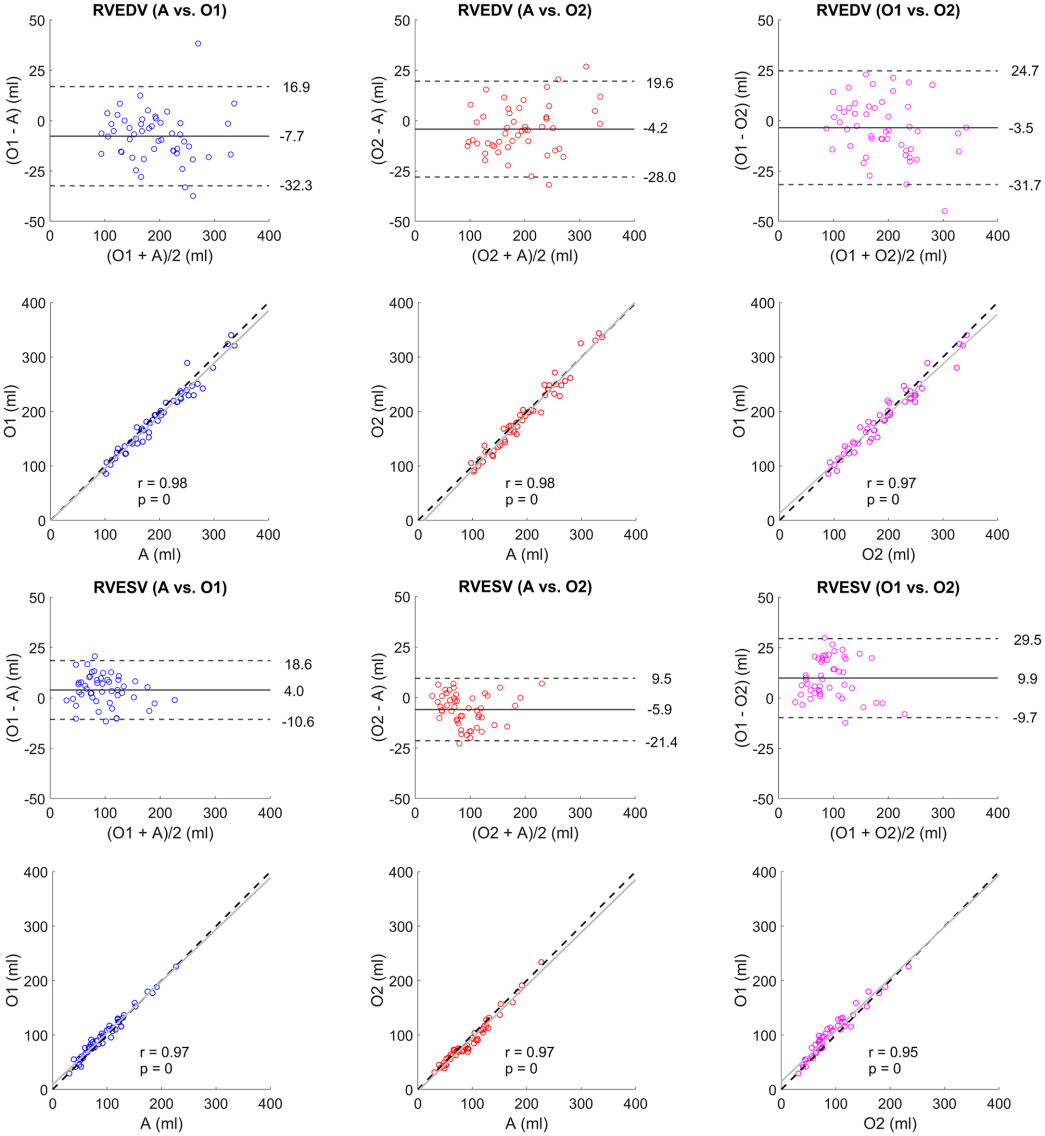
Bland–Altman plots and scatter plots on the clinical validation set. The left column (blue) shows the pipeline's automated (A) delineations vs. Observer 1 (O1), the middle column (red) shows A vs. Observer 2 (O2), and the right column (magenta) shows O1 vs. O2. The top half shows right ventricular (RV) end-diastolic volumes (RVEDV) and the bottom row RV end-systolic volumes (RVESV). The Bland–Altman plots contain bias (full lines) and limits of agreement (± 1.96 SD, dashed lines). The correlation plots contain identity lines (black, dashed lines), least squares lines (grey, full lines), Spearman’s rank correlation coefficients (r) and corresponding *p* values.

The absolute and relative bias between the pipeline and O1 was − 7.7 ± 12.6 ml (− 4 ± 7%) for EDV, 4.0 ± 7.5 ml (4 ± 8%) for ESV, and 4 ± 5% for EF. Bias between the pipeline and O2 was − 4.2 ± 12.1 ml (− 2 ± 6%) for EDV, − 5.9 ± 7.9 ml (− 6 ± 8%) for ESV, and − 2 ± 5% for EF. Bias between observers was − 3.5 ± 14.4 ml (− 2 ± 8%) for EDV, 9.9 ± 10.0 ml (11 ± 11%) for ESV, and − 6 ± 6% for EF, and the pipeline-observer limits of agreement were somewhat narrower than the inter-observer limits of agreement for both ED and ES, with r ≥ 0.95 in both timeframes (Fig. 4). However, the pipeline-observer variability was only significantly different from the inter-observer variability for O1 in ES (*p* = 4.00e−02, n = 50).

For the ACDC testing set, the Dice scores were 0.87 ± 0.09 for ED and 0.80 ± 0.10 for ES, and the Hausdorff distances were 20.18 ± 19.34 for ED and 23.10 ± 16.79 for ES. The absolute and relative bias between the pipeline and the ACDC reference volumes was 6.6 ± 27.2 ml (4 ± 16%) for EDV, − 2.7 ± 15.5 ml (− 3 ± 16%) for ESV, and 5 ± 11% for EF, and the correlation r value was 0.77 for EF as well as 0.85 for EDV and 0.95 for ESV (*p* < 0.0001, n = 50) (Supplementary Fig. S3). For the scan-rescan assessment, the bias between scan and rescan volumes was 6.7 ± 22.8 ml (3 ± 10%) for EDV, 4.8 ± 13.0 ml (4 ± 12%) for ESV, and 0 ± 7% for EF (Supplementary Fig. S4).

For the ratings of the automated delineations on the CVS, the rating A (sufficient for clinical use) dominated, comprising 58% of all subjects. The remaining automated delineations were rated as B (needing minor adjustments), and none were assessed to be in need of major corrections (C). For the test set (TS), 38% of delineations were rated as A, 50% as B, and 12% as C.

The absolute and relative bias between automated and corrected delineations (n = 21) for O1 was − 0.1 ± 6.2 ml (0 ± 3%) for EDV and − 1.4 ± 3.3 ml (− 2 ± 4%) for ESV. For O2, it was 2.9 ± 10.4 ml (2 ± 5%) for EDV and − 0.3 ± 5.3 ml (0 ± 6%) for ESV. For 5 out of 21 ED volumes and 5 out of 21 ES volumes, one observer decreased the volume when the other observer increased it. Plots showing the volumetric changes for the corrections can be seen in Supplementary Fig. S5.

**Figure 5:**
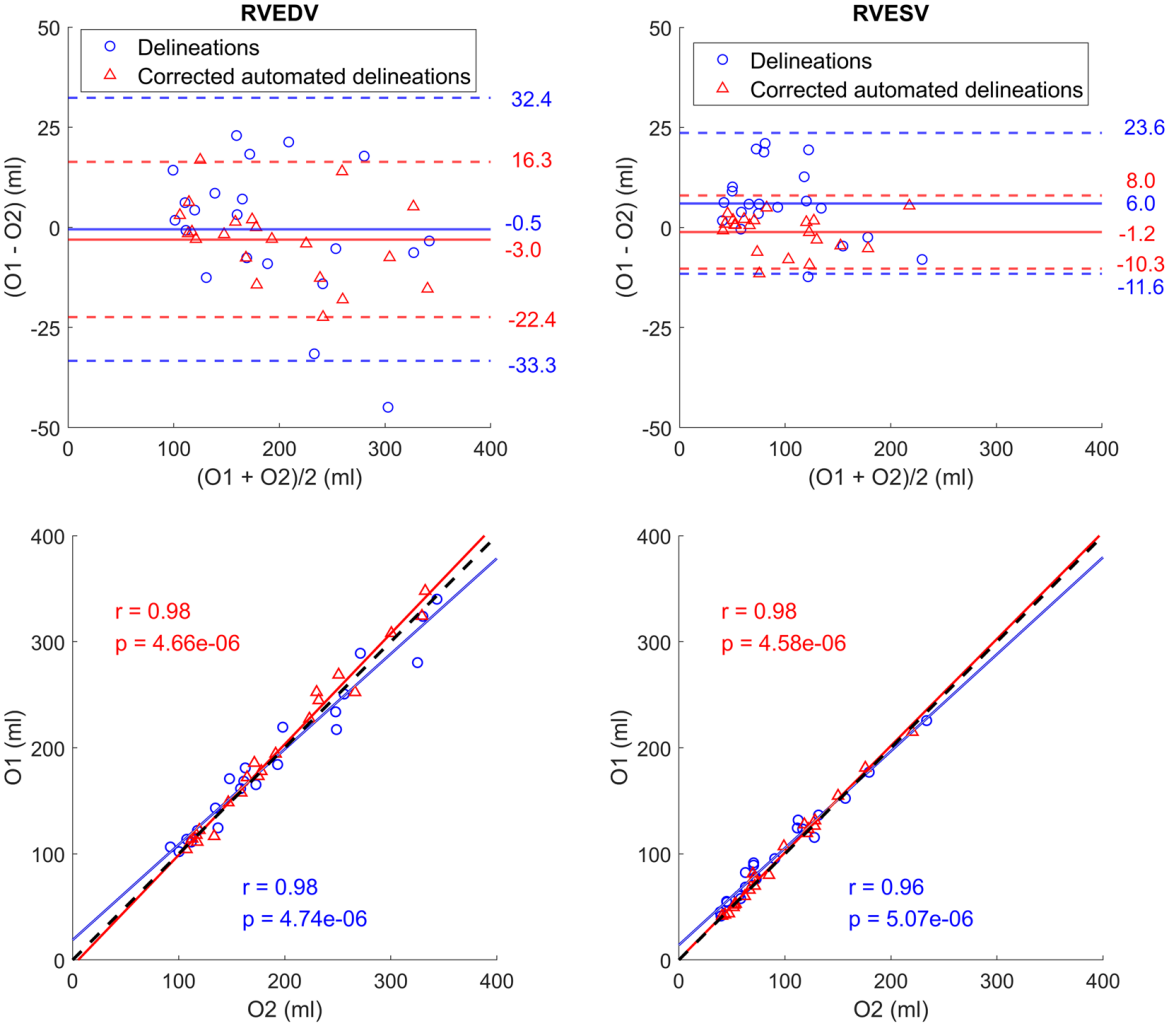
Bland–Altman plots and correlation plots showing the inter-observer variability for manual delineations (blue) and the corrections of delineations from the pipeline (red). To the left are results for end-diastolic volumes (RVEDV) and to the right are results for end-systolic volumes (RVESV). The Bland–Altman plots contain bias (full lines) and limits of agreement (± 1.96 SD, dashed lines). The correlation plots contain identity lines (black, dashed lines), least squares lines (full lines), Spearman’s rank correlation coefficients (r) and corresponding *p* values. Both plots indicate that the inter-observer variability decreased when the pipeline was used, and delineations were corrected.

For the 21 delineations in CVS deemed in need of corrections, the volumetric inter-observer agreement was significantly improved (*p* = 2.23e−02 for ED and *p* = 5.00e−03 for ES, n = 21) (Fig. 5).

For O1, the average manual delineation time was 6 min and 46 s, and the average correction time was 1 min and 38 s (*p* = 5.96e−05, n = 21). For O2, the corresponding times were 5 min and 19 s as well as 2 min and 30 s (*p* = 3.86e−04, n = 18 due to missing data). The mean runtime for the pipeline per subject (in ED and ES) was 2 s on the CVS. The average time reduction (compared to delineating manually) by using pipeline delineations and correcting insufficient ones, was 5 min and 17 s (87% of the average manual delineation time).

## Discussion

We have developed a CNN-based pipeline for RV segmentation and shown its ability to reduce the manual delineation time for obtaining delineations of clinically useful quality according to expert reader ratings. However, the pipeline’s reduced performance when externally validated indicates that time reduction may not be fulfilled for clinics using other RV delineation guidelines.

More than half of the automated delineations on the CVS were rated as of clinically sufficient quality without the need for manual corrections. These delineations were obtained in a matter of seconds using a powerful GPU in a laptop. The remaining delineations were rated as only in need of minor corrections, and the correction time for these was about a third of the manual delineation time. For the TS, 88% of delineations were clinically adequate or in need of minor corrections.

Even if both observers were of CMR level 3, an average of 87% of the average manual delineation time could be saved by using the pipeline. Less experienced observers may require longer manual delineation times, and a larger reduction in delineation time by use of the pipeline could therefore be expected. For example, Caudron et al. (2011) reported mean RV delineation times (for both endocardial and epicardial borders on 60 patients) of 13.4 and 18.9 min for observers with 3 and 1 years of training, respectively^29^. A result of a reduction in delineation time could be that an increased number of patients could be assessed in a day.

We have shown that the use of corrected pipeline delineations was able to decrease the inter-observer variability compared to manual delineations. In a study by Bai et al. (2018), the variability of a CNN-based cardiac segmentation method was on par with the inter-observer variability^15^. Our pipeline-observer variability was lower than the inter-observer variability, although only significantly for O1 in ES (*p* < 0.05). A reason for the substantial ES variability between observers could be that trabeculations may be harder to distinguish from the endocardial wall when the ventricle is contracted. Thus, even if human observers are trained in the same institution and adhere to the same consensus guidelines, they can still vary, especially when delineating the RV in ES. This shows the difficulty of the segmentation problem, and indicates that a common starting point could be helpful for decreasing the inter-observer variability (as seen in Fig. 5). Nonetheless, this also shows the difficulty of validating the performance of automated segmentation methods.

Since the main dataset consisted of delineations from several observers, it is likely that delineation variabilities of a magnitude similar to the one between observers in ES were present. This could have affected the learning process negatively, by the ground truth examples showing contradictory ways of delineating the RV. However, it could also have affected the learning process positively, by allowing the segmentation network to learn a middle ground between the separate observers’ individual opinions on RV delineations. The pipeline-observer bias was lower than between observers for EF and for volumes in ES, and of similar magnitude for volumes in ED, with no observable negative or positive trend.

Larger RVs did not seem to affect the delineation performance of the pipeline in a negative way (Fig. 4), showing the potential to use it in a clinical environment where both healthy cases and pathological cases (with enlarged RVs) may be present. This is important for clinical settings with both pediatric and adult cases. With a decreased inter-observer variability for RV delineations, the possibility to detect true volumetric changes between examinations of a patient at different time points would increase, making CMR an even more robust method for determining changes and trends in pathological states. Moreover, it is important that RV delineations yield replicable volumes close to their true absolute magnitude, since large variations in delineations could mislabel an RV as healthy or pathological^30^ purely due to volumetric errors. The low bias indicates that this might not be a problem for the pipeline. The scan-rescan assessment indicated that inter-scan differences could affect the robustness of the pipeline in some cases. However, no manual reference segmentations were available to determine if the effect would be similar for human observers.

The segmentation network architecture, the 2D U-Net^31^ (see Supplementary Methods S2 for details), is widely used in recent medical image segmentation literature^32^, and its functionality for cardiac segmentation is well described^16^. The novelty of our approach consists of extending this architecture with two additional CNN architectures for robustly pre-processing the CMR images before segmentation, with the purpose of handling the wide variability of CMR data that can be encountered in a clinical setting.

Although the used combination of CNN architectures is new, there exists many previous studies that have presented modifications or additions to segmentation network architectures similar to the one we have used^5–9, 33^. However, unlike this study, most previous studies do not have clinical implementation as the end goal of their method development. Instead, a frequent goal is to create a method that improves upon the segmentation performance of existing deep learning-methods. Performance improvements are commonly assessed using public datasets for training and testing, and evaluations are often performed in a challenge setting. As of November 2022, the 15 RV delineation methods on the Automated Cardiac Diagnosis Challenge post-2017-MICCAI-challenge testing phase leaderboard^23^ presented mean Dice scores in the range of 0.87–0.96 for ED and 0.77–0.90 for ES, mean Hausdorff distances in the range of 8.21–19.20 for ED and 11.65–24.25 for ES, EF correlations (Pearson) in the range of 0.54–0.92 and EDV correlations in the range of 0.92–0.99. The pipeline’s performance on the same dataset was within these ranges, except for ED mean Hausdorff. However, our pipeline was trained using in-house data while the methods on the leaderboard had used the designated ACDC training dataset from the same domain as the testing dataset^23^. This disallows direct comparisons between the learning methodologies. Nonetheless, these results indicate that the trained pipeline can yield delineations that somewhat generalize to segmentation guidelines that differ from those used in its training dataset. Yet, it might not generalize well enough to yield a time reduction as substantial as that observed on the in-house data.

To our knowledge, there is no previous RV segmentation study that has presented the full process from clinical pipeline development to a clinical validation that focuses on the important aspect of time reduction. Existing clinical validation studies^16, 17^ base their analyses on commercial deep learning-based methods and provide limited details on development and function. The pipeline was implemented in the clinical software Segment CMR and made freely available to the research community in the software Segment^18^. We consider this study to be a step forward for the clinical use of deep learning-based RV segmentation methods, by motivating clinicians that still perform fully manual RV segmentations to start using these methods, and by showing clinicians that already use these methods that they can in fact be beneficial, despite the need for corrections.

### Limitations

The delineation quality ratings for defining the quality level of a clinically useful delineation were done by a single observer (O1). Since they are subjective, they may vary between observers, making the presented ratings apply only to the observer that carried them out. However, before the ratings were carried out, the two observers together assessed delineations on 10 separate subjects to ensure a uniform assessment. They also adhered to the same consensus guidelines^22^. Thus, even though the limits of agreement between observers were somewhat wide (Fig. 4), they still have the same general understanding of how the RV should be delineated. It is thus likely that their quality ratings would be similar, and also resemble those of other observers that use the same guidelines. For centers using different guidelines such as excluding trabeculations and papillary muscles from the volumes, the time reduction by using the pipeline was not assessed.

Due to the removal of clinical information during anonymization, it was not possible to assess whether the manual corrections could yield changes in pathological classification. Also, no pathology-specific performance testing could be done. Therefore, no assessment could be made regarding the effect of pathologies on pipeline performance. Nonetheless, our training set included a wide range of volumes and subjects referred to cardiac MR at a large university hospital, making it likely that the pipeline is applicable to pathological cases. Furthermore, the pipeline used short-axis images, and did not make use of transversal (axial) images, which could be a limitation for some institutions. Also, a more robust segmentation model could potentially have been obtained through experimenting more with different types of data augmentation.

We abstained from performing any additional training of our pipeline on any public datasets (e.g. the ACDC training set) because: (a) we used a segmentation network with well-described cardiac segmentation abilities; (b) we believe that the strength of our method can largely be attributed to the width of the training dataset that was used; and (c) the purpose of this study was to validate the clinical usefulness of our pipeline, and since re-training the networks of our pipeline using a different dataset would change the properties of our pipeline, we consider it to be outside the scope of this study.

This study only assessed a reduction in segmentation time compared to fully manual segmentations and did not consider other segmentation methods used in contemporary clinical practice. Moreover, this study showed the possibility to accelerate a single segmentation task. However, these results are not sufficient to determine if the reduced cost from the accelerated segmentation overweighs the potential cost of commercialized deep learning-based segmentation software.

## Conclusion

A deep learning-based clinical pipeline could substantially reduce the time needed to manually obtain RV delineations of clinically sufficient quality, even when considering the occasional need for manual corrections. This indicates that deep learning-based methods can yield right ventricular assessments faster than fully manual analysis in clinical practice, but it remains to be assessed how the observed time reduction would change for clinics adhering to other delineation guidelines.

## Supplementary Information

Supplementary Information.

Supplementary Video S6.

Supplementary Video S7.

Supplementary Video S8.

## Data Availability

Data supporting the findings of this study are available from Skåne University Hospital, but restrictions apply to the availability of these data, which were used under license for the current study, and so are not publicly available. Aggregated data are however available from the authors upon reasonable request and with permission of Skåne University Hospital. The pipeline is implemented in the software Segment (http://segment.heiberg.se) and is freely available for research purposes.

## Abbreviations

RV: Right ventricle
CMR: Cardiac magnetic resonance
RVEDV: Right ventricular end-diastolic volume
RVESV: Right ventricular end-systolic volume
EDV: End-diastolic volume
ESV: End-systolic volume
ED: End diastole
ES: End systole
EF: Ejection fraction
CNN: Convolutional neural network
2D: 2-Dimensional
3D: 3-Dimensional
SD: Standard deviation
O1: Observer 1
O2: Observer 2
CVS: Clinical validation set
TS: Test set
