# Supplementary material for: Deep learning can yield clinically useful right ventricular segmentations faster than fully manual analysis
Source: Sci Rep. 2023 Jan 21;13:1216. doi: 10.1038/s41598-023-28348-y (PMC9867728; doi:10.1038/s41598-023-28348-y)
Supplement: Supplementary file 1 — Supplementary Information. [file 41598_2023_28348_MOESM1_ESM.pdf]

Deep Learning Can Yield Clinically Useful Right Ventricular Segmentations Faster Than Fully Manual Analysis

Julius Åkesson[1,2,], Ellen Ostenfeld[1], Marcus Carlsson[1,3], Håkan Arheden[1], Einar Heiberg[1]*

1 : *Clinical Physiology, Department of Clinical Sciences Lund, Lund University, Skåne University Hospital, Lund, Sweden.*

2 : *Department of Biomedical Engineering, Faculty of Engineering, Lund University, Lund, Sweden.*

3 : *Laboratory of Clinical Physiology, National Heart, Lung and Blood Institute, National Institutes of Health, Bethesda, Maryland, USA.*

*: Correspondence: *julius.akesson@med.lu.se*

Supplementary Methods S1: Main dataset characteristics

The main dataset was assembled by combining short-axis CMR examinations from clinical scans collected between 2019 and 2020 and research scans from 2004-2020.

The included scans were intended to contain a wide range of pathologies, vendors, and image sizes, to enable the development of a pipeline that is robust towards these attributes. Only 5 % of the total number of scans in the main dataset were known to be from healthy subjects. For the subjects that were from clinical practice, no statistics on underlying pathologies could be made, due to full anonymization being required before inclusion to the study. As a surrogate, we assessed the distribution of RVEDV in the training set (Supplementary Fig. S1). This analysis showed that there were examinations both within and outside the normal ranges for men and women [1].

Many previous cardiac segmentation methods have used data from a subset of the UK Biobank [2] [3] or the ACDC challenge [4–6] for development and evaluation. Both the UK Biobank and the ACDC dataset contain single site and single vendor data. Our data were included over a 16-year period

using three different vendors. Our dataset (1693 scans from 1434 subjects) is larger than the ACDC dataset (150 subjects [7]), but smaller than the UK Biobank dataset (over 5000 subjects [1]). The ACDC dataset contains five sub-groups of subjects, of which four are pathological [7]. The observed wide range of RV volumes, and the fact that 81 percent of the examinations in the main dataset came from the primary cardiac MR facility at a university hospital, indicates that the main dataset may contain a wider range of pathologies than the ACDC dataset.

The median size of a 3D timeframe in the training set was 200 x 195 x 17 (after re-sampling to the median pixel spacing of the training set). The size of the first dimension ranged from 102 to 463 pixels, the size of the second dimension ranged from 94 to 468 pixels and the size of the third dimension ranged from 10 to 30 slices. This indicates that a variety of used scanners, protocols, or pre-processing techniques (including manual cropping) had been used. All scans were acquired using bSSFP. In the main dataset, the temporal resolution ranged between 0.0106 and 0.0916 seconds, and the spatial resolution ranged between 0.5013 and 3.5156 millimeters.

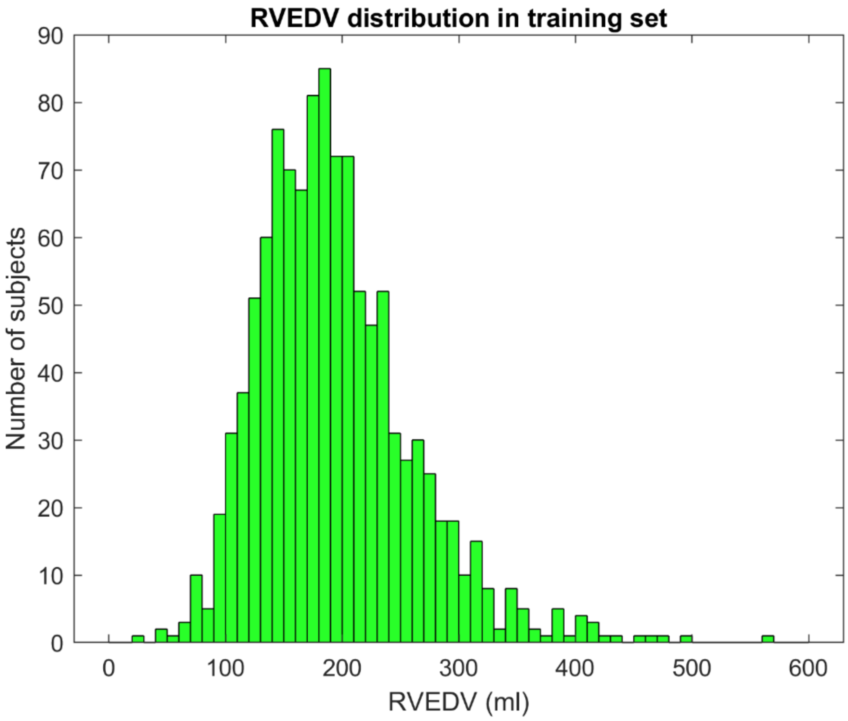

Supplementary Figure S1: Histogram showing the distribution of right ventricular end-diastolic volume (RVEDV) in the training set.

Supplementary Methods S2: Network architecture details

All networks were developed using the Deep Learning Toolbox in MATLAB R2019a and R2021a (Natick, Massachusetts: The MathWorks Inc.; 2019 and 2021). A checklist [8] was used to ensure sufficient information was presented.

Details on the slice selection network

The used architecture for the slice selection network was the classification architecture (Darknet-19) presented by Redmon and Farhadi (2016) [9]. This architecture consists of 19 convolutional layers (with varying amounts of 3 x 3 kernels) and five max-pooling layers [9]. The last convolutional layer of this architecture was modified to carry two convolutional kernels instead of 1000 (as in the original architecture), making it eligible for binary classification. The output was given by a softmax activation function. This exact modification had previously been implemented for the LV segmentation pipeline in Berggren et al. (2020) [10].

Details on the RV center point detection network

The center point detection network was based on the exact same network architecture as the slice selection network (i.e. Darknet-19) [9]. The only difference from the slice selection network was the use of a ReLU function as the output activation function.

Details on the segmentation network

The segmentation network architecture was a 2D U-Net [11]. The implementation of this architecture was generated by using the MATLAB R2019a function unetLayers with the input parameters 'imageSize' = 256, 'EndoderDepth' = 4 and 'numClasses' = 2. The input image size 256 x 256 was chosen to be able to contain the maximum dimensions of the RV found in the training set with robustness towards possible inaccuracies in the RV center point detection. The output layer was replaced by a layer generated by the MATLAB function dicePixelClassificationLayer.

Supplementary Methods S3: CNN training details

Statistical metrics for evaluating individual CNNs

Different metrics were used for the individual performance evaluations of the different CNN models. For the slice selection network, the classification accuracy was used. For the center point detection network, the mean squared error (MSE) of the regression output was used. The segmentation network was evaluated using the Dice score (Sørensen-Dice coefficient [12]).

General training details

All training procedures were performed on either a single NVIDIA TITAN RTX GPU or a single NVIDIA GeForce RTX 3090 GPU. During the grid search five-fold cross validation (5-FCV) for hyperparameter optimization, the best combination of learning rate, batch size, and L2-regularization was sought. For all hyperparameters not mentioned in the following sections, the preset values in the Deep Learning Toolbox in MATLAB R2019a were used. For the 5-FCV, the used stopping criteria was a limited number of epochs (ten). To monitor the training processes during the 5-FCV, the model performance was evaluated on the test fold for each epoch during training. No overfitting was observed. During model selection, several types of image augmentation were tested, including rotation, translation, and Gaussian noise. However, this yielded no apparent effect on the performance of any of the networks during the 5-FCV. Therefore, no augmentation was applied.

During the final training (after model selection), each network was trained for 100 epochs. In this case, a small validation set (5 % of the training set) was used to monitor overfitting.

Training the slice selection network

The slice selection network was trained using all slices from each 3D timeframe of the training set. This implementation took images of 256 x 256 pixels as its input. Thus, images were reshaped using bilinear interpolation before insertion (for both training and inference). The ground truth training

labels for binary classification were automatically generated by labeling each slice that had an existing reference delineation as 1, and the remaining slices as 0. The weights of the trainable filters of the network were initialized using Glorot initialization [13]. The network was trained using a Stochastic Gradient Descent with Momentum optimizer and a cross-entropy loss function. The used hyperparameters (found through grid search 5-FCV) were a learning rate of 0.001, a batch size of 30 and an L2 regularization factor of 0.01.

Training the center point detection network

The center point detection network was trained using all slices in the training set that had an existing reference delineation. The training data for this network was generated by pairing each such 2D slice with a ground truth center point that was calculated as the most central pixel of the foreground RV object in the reference delineation mask. The trainable kernel parameters of this network were initialized using Glorot initialization [13]. This network was trained using an Adam optimizer [14] and a mean squared error loss function. The hyperparameters (found during grid search 5-FCV) were a learning rate of 0.0001, a batch size of 10 and an L2 regularization factor of 0.0001.

Training the segmentation network

This segmentation network was also trained exclusively on slices with existing reference delineations. For the training, an Adam optimizer [14] and a generalized Dice loss function (suitable for handling class imbalances between background and foreground pixels [15]) were used. Learnable parameters were initialized using Glorot initialization [13].

The used hyperparameters (found through Grid search 5-FCV) was a learning rate of 0.0001, a batch size of 20 and an L2 regularization factor of 0.0001.

Supplementary Results S4: Pipeline performance evaluation on the test set

Separate performance evaluations of each of the final three models were carried out on the unrefined test set. The slice selection network obtained an accuracy of 0.93 (when tested on the 242 patients from the unrefined test set that had no slices with missing delineations). The RV center point detection network obtained a mean (across slices) MSE of 25.35. The segmentation network obtained a mean (across slices) Dice score of 0.90.

Performance evaluations of the full pipeline were carried out on the refined test set (TS). The quality-controlled TS delineations had a mean EDV of 193.5 ± 65.2 ml and ESV of 101.8 ± 53.9 ml. The bias between volumes from automated and reference delineations (and their percentages of the mean reference volumes) was -8.8 ± 21.7 ml (-5 ± 11 %) for EDV and -8.2 ± 16.1 ml (-8 ± 16 %) for ESV. Bland-Altman and correlation plots are provided in Supplementary Figure S2. The pipeline obtained a mean Dice score (across timeframes) of 0.87 on the TS, with mean Dice scores of 0.89 for ED and 0.87 for ES. The difference between these results and the results on the CVS may be due to the varying quality and origin of the reference delineations in the TS.

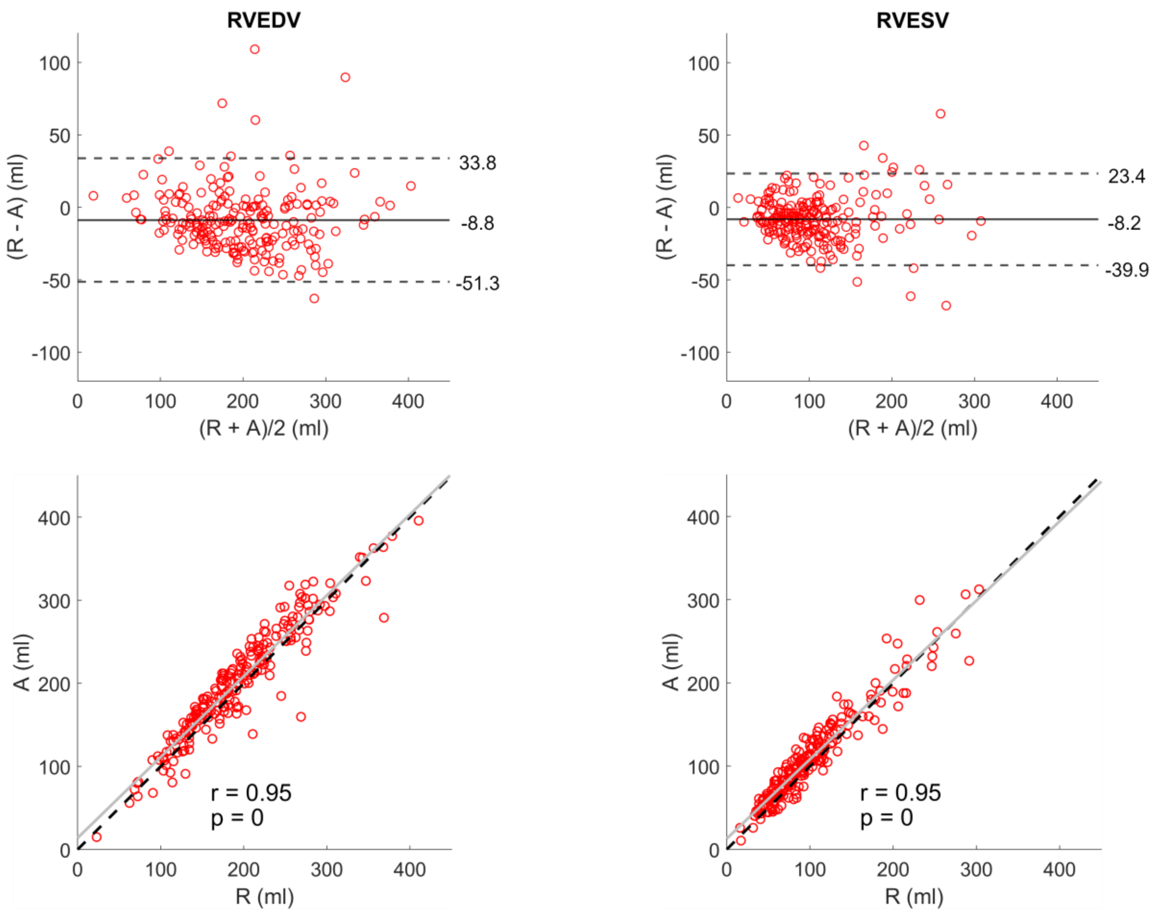

Supplementary Figure S2: Bland-Altman and correlation plots between the pipeline's automated (A) and the reference (R) RVEDV (left column) and RVESV (right column) on the refined test set (TS). The Bland-Altman plots contain bias (full lines) and limits of agreement (± 1.96 SD, dashed lines). The correlation plots contain identity lines (black, dashed lines), least squares lines (grey, full lines), Spearman's rank correlation coefficients (r) and corresponding p-values.

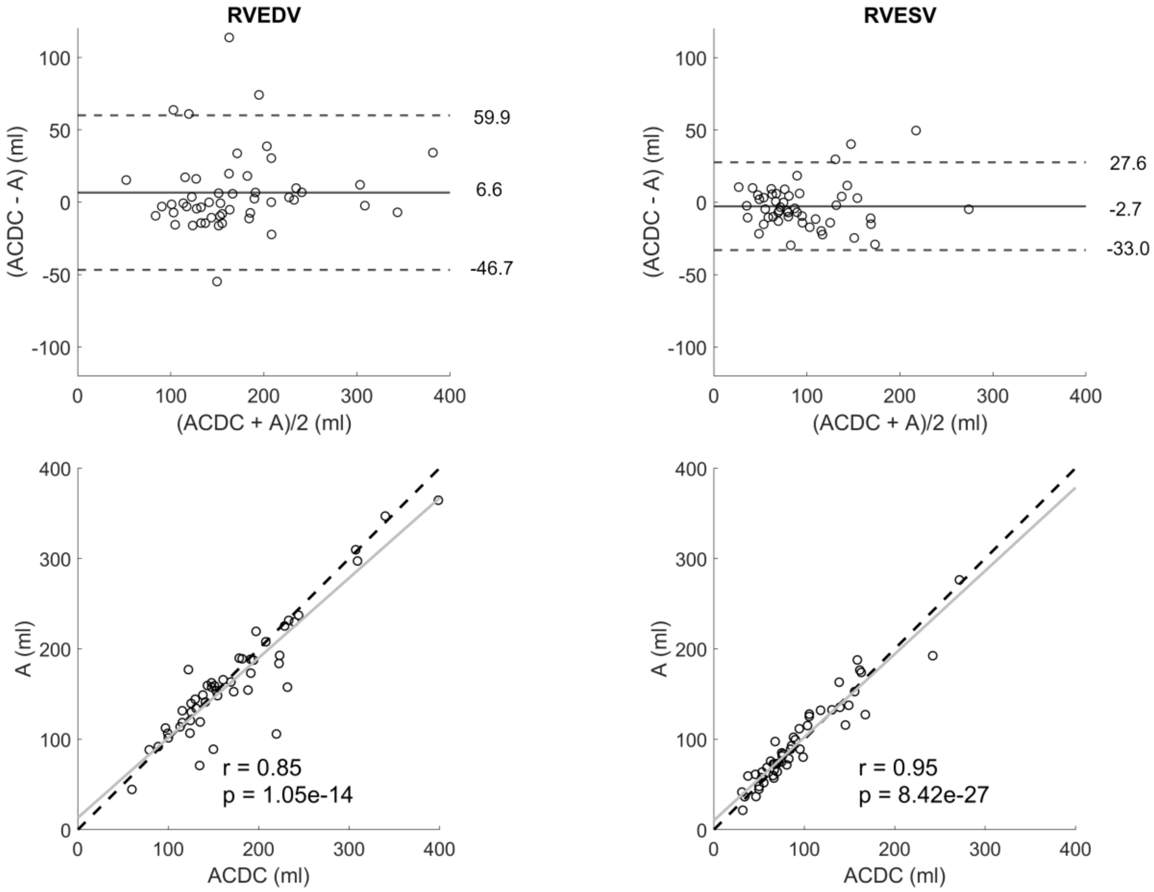

Supplementary Figure S3: Bland-Altman and correlation plots between the pipeline's automated (A) and the ACDC reference RVEDV (left column) and RVESV (right column) on the ACDC test set [7]. The Bland-Altman plots contain bias (full lines) and limits of agreement (± 1.96 SD, dashed lines). The correlation plots contain identity lines (black, dashed lines), least squares lines (grey, full lines), Spearman's rank correlation coefficients (r) and corresponding p-values.

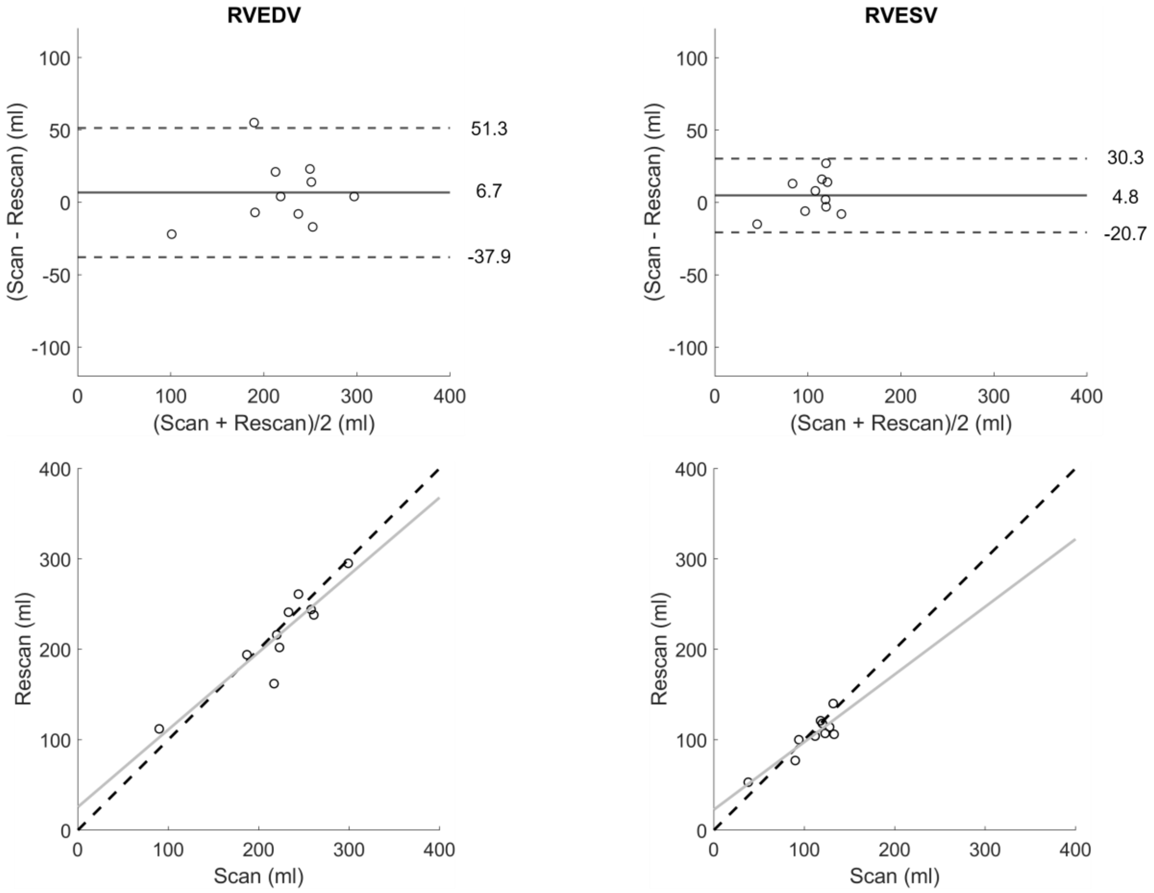

Supplementary Figure S4: Bland-Altman and correlation plots between scan and rescan RVEDV (left column) and RVESV (right column) on the 10 scan-rescan subjects. The Bland-Altman plots contain bias (full lines) and limits of agreement (± 1.96 SD, dashed lines). The correlation plots contain identity lines (black, dashed lines) and least squares lines (grey, full lines).

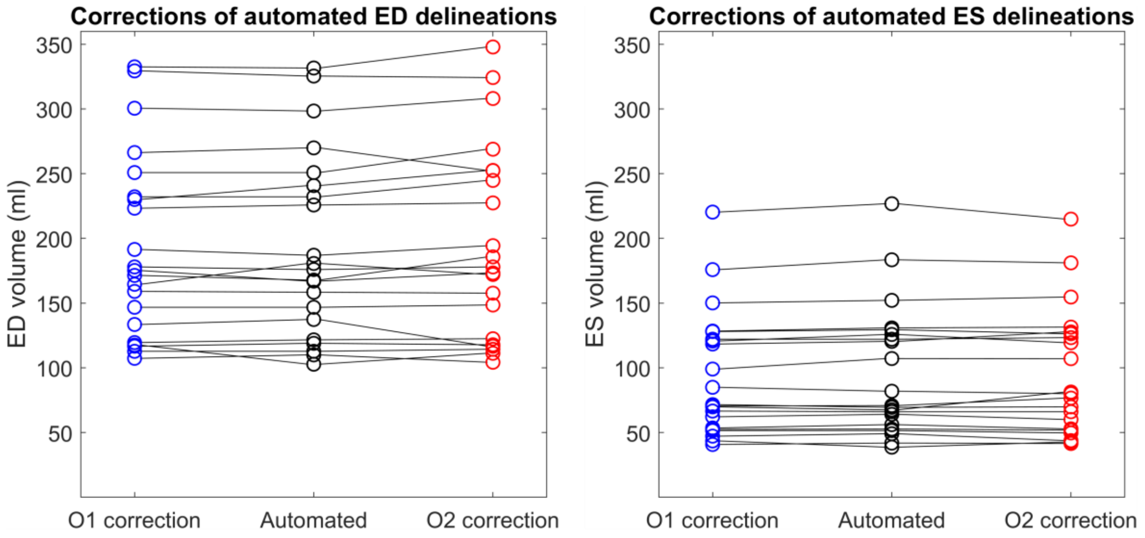

Supplementary Figure S5: The volumetric changes yielded by manual corrections of automated delineations by Observer 1 (O1) and Observer 2 (O2). The black markers are the volumes from the automated delineations, while the blue markers are the volumes from O1's corrections and the red markers are the volumes from O2's corrections. The black lines connect volumes from the same subject. As expected for minor corrections, corrected volumes changed only slightly compared to the automated volumes. In most cases, the volumes were similar between observers after correction.

Supplementary Methods S5: Examples of subjective delineation quality ratings

Examples of delineations in end diastole and end systole for the three possible subjective delineation quality ratings performed by Observer 1 can be seen in Supplementary Videos S6-S8 online. Supplementary Video S6 shows an example of rating A, for which the automated delineation was of sufficient quality for clinical use. Only the automated delineation generated by the pipeline is shown (magenta). Supplementary Video S7 shows an example of rating B, for which the automated delineation needed minor corrections. This shows the automated delineation generated by the pipeline (magenta, dotted) as well as corrections of this delineation by Observer 1 (red, full) and Observer 2 (green, dashed). Supplementary Video S8 shows an example of rating C, for which the automated delineation needed major corrections. This shows the automated delineation generated by the pipeline (magenta, dotted), as well as a delineation noted "Observer" (cyan, dotted), referring to a quality-controlled delineation from the main dataset performed by an unknown observer.

Supplementary Legends for Supplementary Videos S6, S7 and S8

Supplementary Video S6: An example of rating A, for which the automated delineation was of sufficient quality for clinical use. This shows the automated delineation generated by the pipeline (magenta) in end diastole (ED, left) and end systole (ES, right).

Supplementary Video S7: An example of rating B, for which the automated delineation needed minor corrections. This shows the automated delineation generated by the pipeline (magenta, dotted) as well as corrections of this delineation by Observer 1 (red, full) and Observer 2 (green, dashed) in end diastole (ED, left) and end systole (ES, right).

Supplementary Video S8: An example of rating C, for which the automated delineation needed major corrections. This shows the automated delineation generated by the pipeline (magenta, dotted), as well as a delineation denoted "Observer" (cyan, dotted), referring to a quality-controlled delineation from the main dataset performed by an unknown observer, in end diastole (ED, left) and end systole (ES, right).

References

1. Petersen, S. E. *et al.* Reference ranges for cardiac structure and function using cardiovascular magnetic resonance (CMR) in Caucasians from the UK Biobank population cohort. *Journal of Cardiovascular Magnetic Resonance* **19**, 1–19 (2017).

2. Chen, C. *et al.* Improving the generalizability of convolutional neural network-based segmentation on CMR images. *Frontiers in cardiovascular medicine* **7**, 105 (2020).

3. Bai, W. *et al.* Automated cardiovascular magnetic resonance image analysis with fully convolutional networks. *J Cardiovasc Magn Reson* **20**, 65 (2018).

4. Isensee, F. *et al.* Automatic Cardiac Disease Assessment on cine-MRI via Time-Series Segmentation and Domain Specific Features. in *Statistical Atlases and Computational Models of the Heart. ACDC and MMWHS Challenges* (eds. Pop, M. et al.) vol. 10663 120–129 (Springer International Publishing, 2018).

5. Calisto, M. B. & Lai-Yuen, S. K. AdaEn-Net: An ensemble of adaptive 2D–3D Fully Convolutional Networks for medical image segmentation. *Neural Networks* **126**, 76–94 (2020).

6. Simantiris, G. & Tziritas, G. Cardiac MRI Segmentation With a Dilated CNN Incorporating Domain-Specific Constraints. *IEEE J. Sel. Top. Signal Process.* **14**, 1235–1243 (2020).

7. Bernard, O. *et al.* Deep Learning Techniques for Automatic MRI Cardiac Multi-Structures Segmentation and Diagnosis: Is the Problem Solved? *IEEE Trans. Med. Imaging* **37**, 2514–2525 (2018).

8. Mongan, J., Moy, L. & Kahn, C. E. Checklist for Artificial Intelligence in Medical Imaging (CLAIM): A Guide for Authors and Reviewers. *Radiology: Artificial Intelligence* **2**, e200029 (2020).

9. Redmon, J. & Farhadi, A. YOLO9000: better, faster, stronger. in *Proceedings of the IEEE conference on computer vision and pattern recognition* 7263–7271 (2017).

10. Berggren, K. *et al.* Multiple Convolutional Neural Networks for Robust Myocardial Segmentation. in *In proceedings of SSBA 2020* (2020).

11. Ronneberger, O., Fischer, P. & Brox, T. U-Net: Convolutional Networks for Biomedical Image Segmentation. in *Medical Image Computing and Computer-Assisted Intervention – MICCAI 2015* (eds. Navab, N., Hornegger, J., Wells, W. M. & Frangi, A. F.) vol. 9351 234–241 (Springer International Publishing, 2015).

12. Bell, D. & Moore, C. Dice similarity coefficient. in *Radiopaedia.org* (Radiopaedia.org, 2020). doi:10.53347/rID-75056.

13. Glorot, X. & Bengio, Y. Understanding the difficulty of training deep feedforward neural networks. in *Proceedings of the thirteenth international conference on artificial intelligence and statistics* 249–256 (JMLR Workshop and Conference Proceedings, 2010).

14. Kingma, D. P. & Ba, J. Adam: A method for stochastic optimization. *arXiv preprint arXiv:1412.6980* (2014).

15. Milletari, F., Navab, N. & Ahmadi, S.-A. V-net: Fully convolutional neural networks for volumetric medical image segmentation. in *2016 fourth international conference on 3D vision (3DV)* 565–571 (IEEE, 2016).
