# Supplementary figures and images for: Deep learning can yield clinically useful right ventricular segmentations faster than fully manual analysis
Source: Sci Rep. 2023 Jan 21;13:1216. doi: 10.1038/s41598-023-28348-y (PMC9867728; doi:10.1038/s41598-023-28348-y)

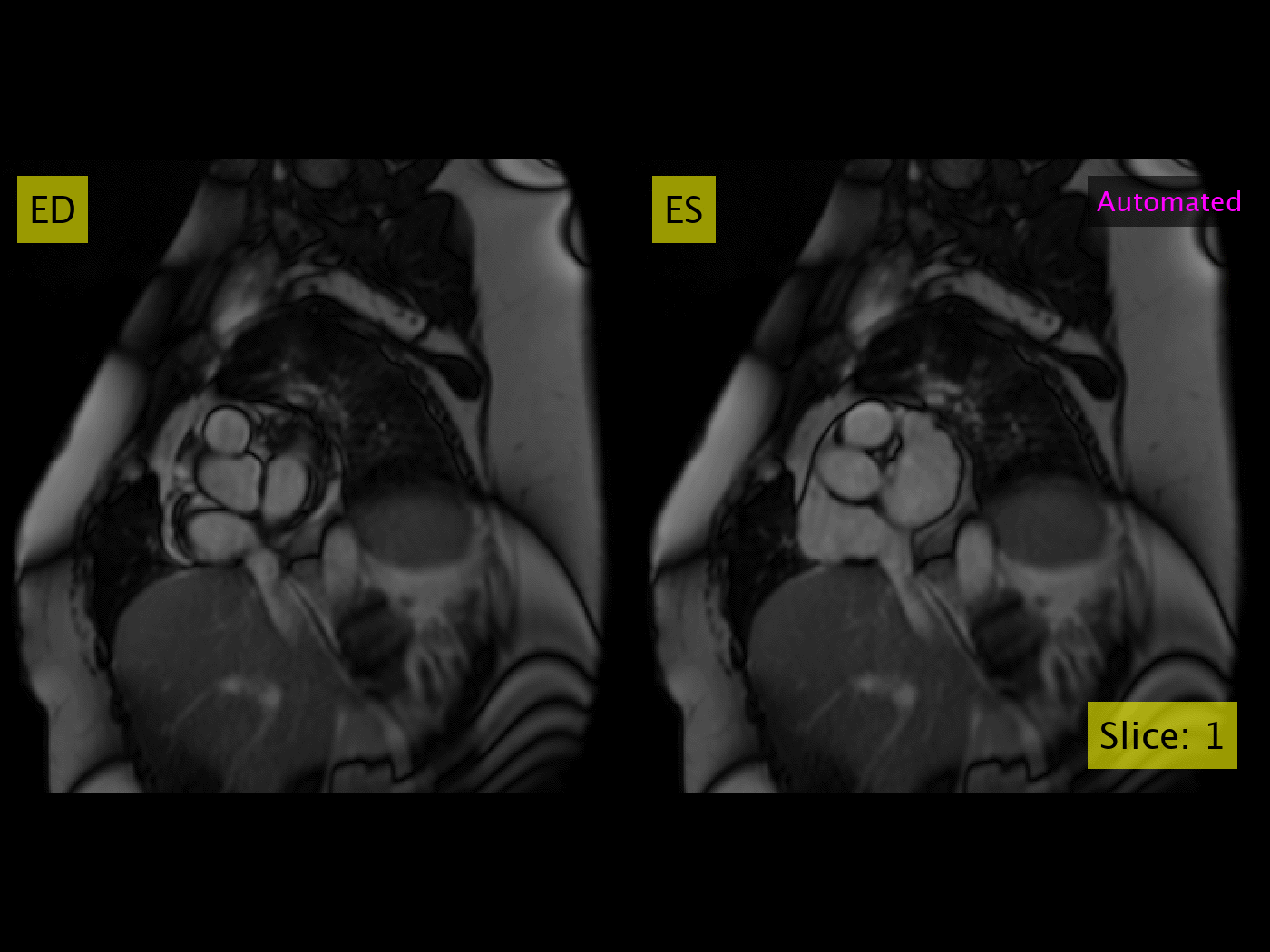

Supplement: Supplementary file 2 — Supplementary Video S6. [file 41598_2023_28348_MOESM2_ESM.gif]

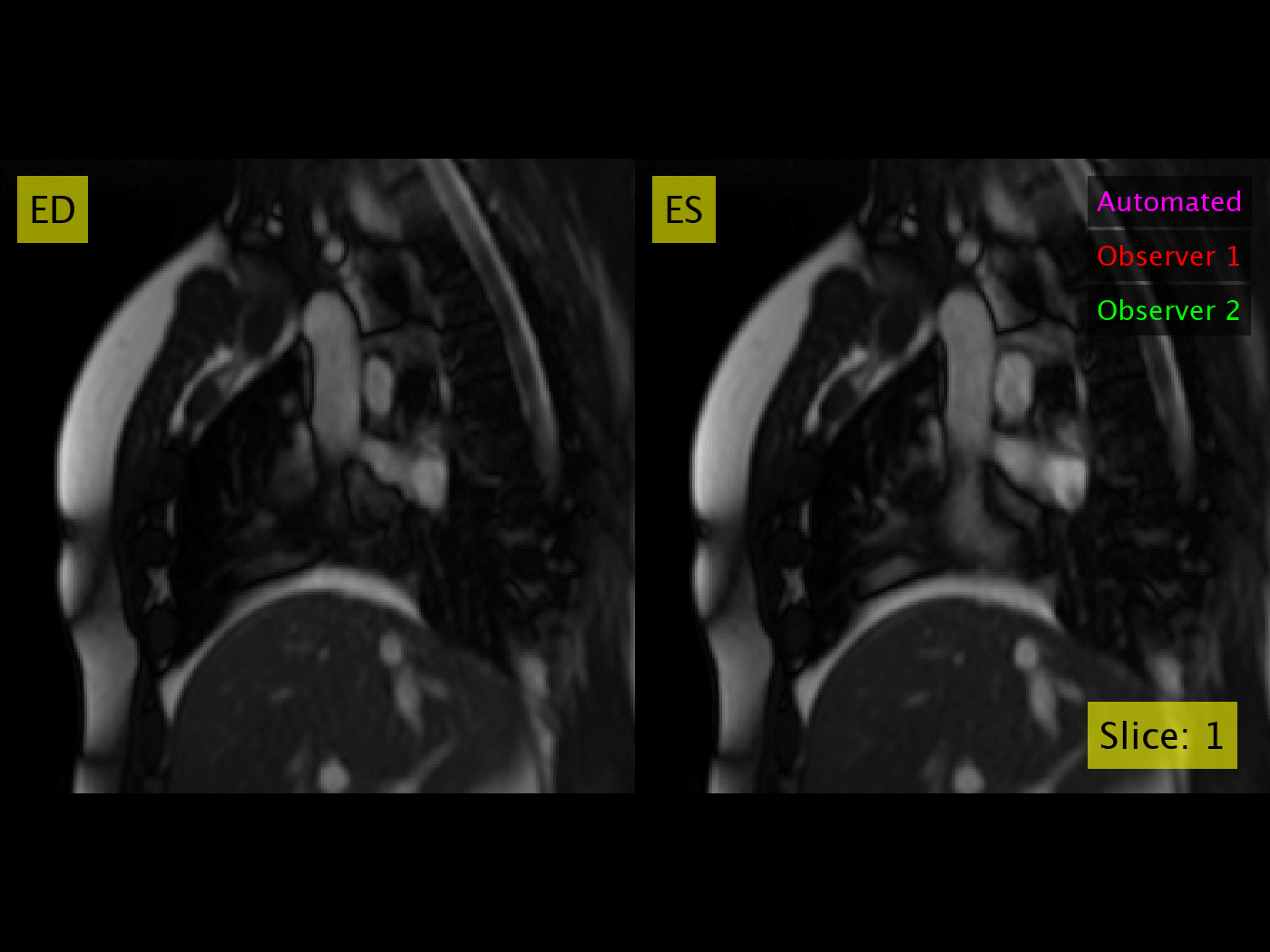

Supplement: Supplementary file 3 — Supplementary Video S7. [file 41598_2023_28348_MOESM3_ESM.gif]

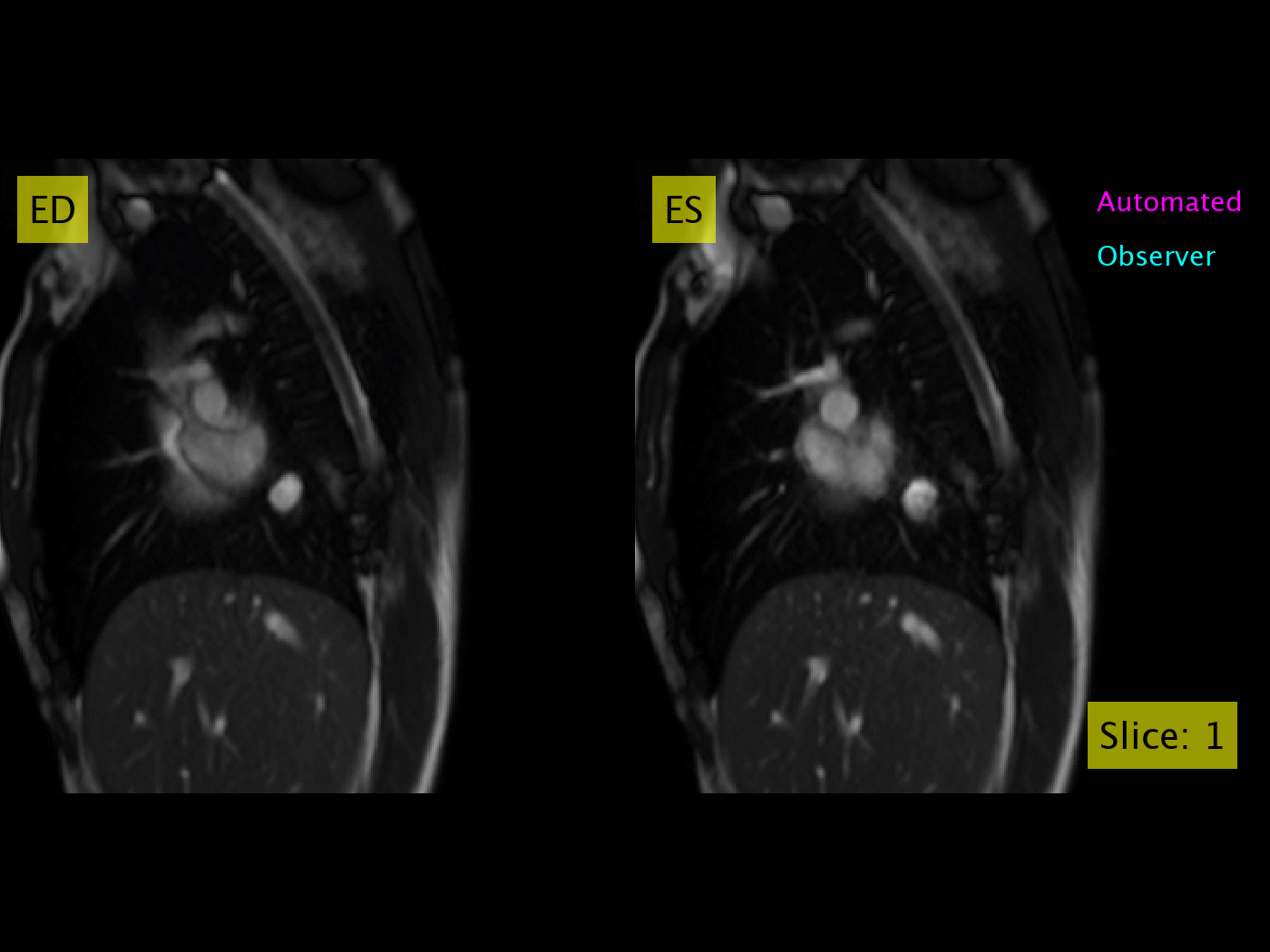

Supplement: Supplementary file 4 — Supplementary Video S8. [file 41598_2023_28348_MOESM4_ESM.gif]
